# Sociodemographic, lifestyle, behavioral, and parental factors associated with sugar-sweetened beverage consumption in children in China

**DOI:** 10.1371/journal.pone.0261199

**Published:** 2021-12-10

**Authors:** Haijun Guo, Dung Phung, Cordia Chu

**Affiliations:** Centre for Environment and Population Health, Griffith University, Nathan, Australia; Universita degli Studi di Brescia, ITALY

## Abstract

**Objective:**

Evidence shows sugar-sweetened beverage (SSB) consumption is a risk factor for obesity and non-communicable diseases (NCDs) in children. Investigating the influential profiles, which have been examined insufficiently, will help to inform the reduction of SSB consumption. The present research examines the current trend in SSB consumption and associated factors among children in China, in order to inform policy development.

**Methods:**

Secondary data was extracted from China’s Health and Nutrition Survey (CHNS; 2004, 2006, 2009, and 2011), a repeated cross-sectional research, and a Chi-squared test was applied to compare SSB consumption in the last year, queried by social demographical, `environmental, behavioral, and parental factors. Multilevel mixed-effects logistic regression was employed to examine the trend and effects of the multiple factors.

**Results:**

A total of 6015 Chinese children aged 6–17 years were investigated. From 2004 to 2011, the percentage of SSB consumption in children increased from 72.6% to 90.3%. The prevalence in urban areas was higher than the prevalence in rural areas, higher in high schools than primary and middle schools, higher in east coast affluent provinces than other provinces, and higher in high-income households than low-income households. Other associated factors include children’s fast food and salty snacks preference, level of physical activity, sedentariness, and parental education. The strongest association with SSB consumption in children was the mother’s SSB consumption (adjusted odds ratio: 5.54, 95% CI: 3.17–9.67).

**Conclusion:**

Children’s SSB consumption has increased significantly in China, and is associated with socio-economic, demographic, level of physical activity, food preference, and parental factors. Future strategies aimed at reducing SSB consumption among children need to consider these factors.

## Introduction

Obesity plays a key role in the pandemic of non-communicable diseases (NCDs), with the highest mortality and morbidity worldwide, and imposes a heavy burden on healthcare systems [1, 2]. Research shows the prevalence of childhood obesity has increased by 8.3% annually from 1981 to 2010 [3–5], causing a higher likelihood of obesity in adulthood. Accumulating research illustrates childhood obesity is increasing dramatically in developing countries: For example, China has seen a significant increase in overweight and obesity in children [6–10]. The soaring prevalence of obesity and NCDs is recognized as a consequence of an interaction of multiple factors, such as diet shifting, eating out more frequently, increased intake of cooking oils, reduced physical activity, and increased sedentary behavior. Among these, an excess of free sugar in the diet has been identified as one of the most significant factors [3]. The main source of dietary free sugar is SSBs, especially in children [11, 12].

Research has shown that SSB sales have increased during the last two decades, particularly in low-and middle-income countries: The figures increased from 43.4 liters per person in 2001 to 65.3 liters per person in 2014 [13]. China has experienced a sharp increase in SSB sales [14], but evidence regarding the frequency of SSB consumption in the population remains lacking, in particular the trend of SSB consumption by children during the last two decades is missing.

Researchers have claimed that social and environmental factors affect SSB consumption, such as economic status, water fountains in the community, advertising, marketing, in addition to the dietary behaviors and physical activities, and parental influence has been found to be highly associated with SSB consumption in children [15–19]. These findings are mainly drawn from cross-sectional studies [20], and the historical influence of these previously examined factors remains unclear, especially in countries such as China that are undergoing rapid socio-economic change. Moreover, previous studies have examined the effects of associated factors in small-scale studies, focusing only on sex, age, and residency while ignoring a wide range of socio-economic and lifestyle factors. This information gap presents a challenge for policy makers in developing effective strategies to reduce SSB consumption in children. Thus, this research aims to examine the trend and various associated factors of SSB consumption among children in China.

## Methods

### Setting and participants

We extracted the data from the CHNS, which was a population-based, multistage, cluster-random sampling survey. From 1989 to 2015, nine rounds of nutrition, health behavior, health status, and sociodemographic data was collected. Households were the basic sample units from rural and urban areas in nine provinces, including Heilongjiang, Liaoning, Shandong, Henan, Jiangsu, Hubei, Hunan, Guizhou, and Guangxi. All members of each household were investigated. A detailed description of the survey design and procedures has been published elsewhere [21, 22].

We selected participants aged 6–17 years to analyse their SSB consumption in 2004, 2006, 2009, and 2011 [22]. The data was openly accessed on the CHNS official website. The ethical clearance of CHNS was approved by the IRB of the National Institute for Nutrition and Food Safety, China Center for Disease Control and Prevention, and University of North Carolina at Chapel Hill [21, 22]. The current research was fully approved by the Griffith University Human Research Ethics Committee. Written informed consent was obtained from all participants, data was fully anonymized, and no identifiable information was collected.

### Variables measured

Questionnaires were developed and adapted by CHNS to measure the frequency of beverage consumption and the potential associated factors. All these questionnaires had been documented and described on the CHNS website [22].

#### Beverage consumption

SSB consumption was measured through two questions that were included in the CNHS: “In the last year, did you drink soft drinks or sugared fruit drinks?” and “How often did you drink soft drinks or sugared fruit drinks?” For the first question, the potential answers were ‘No’, ‘Yes’, and ‘Unknown’. The children who answered ‘Yes’ were required to answer the second question, and the potential answers were: ‘almost every day’, ‘3–4 times a week’, ‘1–2 times a week’, ‘1–2 times a month’, ‘no more than once a month’, and ‘unknown’. Because SSB consumption showed a highly skewed distributed and was largely reported as zero in previous research [23], we defined the answer ‘No’ for the first questions as ‘0 times a week’ in the final variable; ‘1–2 times a week’, ‘1–2 times a month’, and ‘no more than once a month’ for the second question as ‘0–2 times a week’; ‘almost every day’, ‘3–4 times a week’ for the second question as ‘>2 times a week’. The ‘unknown’ for the first and second questions were defined as missing in the final variable, because they did not provide enough information to assess SSB consumption in this research.

#### Associated factors

We selected the potential associated factors according to literature and theoretical plausibility. Age, sex, residency, education level, and provincial categories were demographic factors frequently used by previous research [24]. We selected household income to evaluate the influence of economic status on SSB consumption [24], preference for fast food, salty snack, vegetable and fruit indicating to evaluate the influence of dietary habit [25–27], physical exercise and sedentary activity to evaluate the influence of lifestyle [19, 28], and parental education and SSB consumption to evaluate the parental influence [29].

We defined those associated factors as age (categorized into two age groups: 6–11 years or 12–17 years), sex (boy or girl), education (primary school, middle school, or high school), residency (rural or urban), and provincial categories (northeast, east coastal, central, south, or municipality). Household income per year was categorized into three groups by quartile (low: 0 to quartile 25, medium: quartile 25 to quartile 75, high: over quartile 75). Food preferences were binary variables (yes or no). Performing physical exercises out of school and in school were respectively classified into two groups (yes or no). Sedentary activity was defined as low, medium, and high according to the homework plus screen time (less than 2 hours, 2 to 4 hours, and more than 4 hours). Father and mother’s highest education level were classified into three groups: primary and below, middle school, and graduate and postgraduate. Whether or not father and mother reported SSB consumption in the last year was also analyzed as two dichotomous factors.

### Statistical analysis

We used a Chi-square test to compare the percentages of reported SSB consumption queried by factors among children. The Mantel-Haenszel chi-square was used if the factors were in ordinal scales.

Given that participants might be repeatedly investigated across the four survey years, the multilevel mixed-effects logistic regression were applied to determine the adjusted odds ratios (AOR) and 95% confidence intervals (CI) of the occurrence of SSB consumption as the dependent variable (Y) and dummy variables for children’s factors referring to demographic, economic, food preferences, physical activity, sedentary activity, and parental influences as independent variables. We chose participants as the random-effect intercept in the regression model. The full model was provided, and the goodness-of-fit was illustrated with Akaike information criterion (AIC). All analyses were conducted in SAS, version 9.4 (SAS Institute Inc., Cary, NC, United States). The statistical significance level was set at 0.05.

## Results

### Characteristics of participants

In total, 6015 children aged 6–17 years, including 3198 boys and 2817 girls, were involved. Four rounds of survey were used with sample sizes of 1725 (2004), 1385 (2006), 1241 (2009), and 1663 (2011) respectively (Table 1). Younger children aged 6–11 years had a similar sample size (51.0%) compared with those aged 12–17 years (49.0%), while the sample size of rural children (69.6%) was more than twice the urban sample size (30.4%), and boys (53.2%) outnumbered girls (46.8%).

**Table 1:** Characteristics of participants stratified by age, sex and residency.

	2004	2006	2009	2011
Age				
6–11	732	715	656	966**
12–17	993	671	585	697
Sex				
Boys	919	735	690	854
Girls	806	651	551	809
Residency				
Urban	484	398	341	608**
Rural	1241	988	900	1055

* Chi-Square test: p<0.05;

** Chi-Square test: p<0.01.

### Sugar-sweetened beverage consumption among children in China from 2004 to 2011

In total, 81.2% of children reported they had consumed SSBs in the last year during 2004–2011, and 16.8% of them consumed SSBs more than twice a week in the same period. The prevalence of SSB consumption among children increased significantly during the research period (Mantel-Haenszel χ^2^ = 185.6, P<0.01). In 2004, 14.2% of children consumed SSBs two times a week, which increased to 21.8% in 2011, while 27.4% of the children who did not consume SSBs decreased to only 9.7% during the same period.

The result of bivariate analysis (Table 2) shows that differences of the SSB consumption between age groups in each survey year was not significant (P>0.05). A higher percentage of boys (92.0%) consumed SSBs than girls (88.5%) in 2011 (P<0.01), while a higher percentage of urban children consumed SSBs than rural children in 2004 (84.0% vs. 68.1%), 2006 (83.0% vs. 72.1%) and 2009 (89.9% vs. 86.0%), and the differences were statistically significant. High school students tended to consume more SSBs, in particular 32.0% of high school students consumed SSBs more than twice per week, which is significantly higher than that of primary school students (19.4%) and middle school students (27.1%) (Mantel-Haenszel p<0.01) in 2011. The children living in east coast provinces had a higher prevalence of SSB consumption than for other provinces in 2004, 2006, and 2011 (P<0.01). As the household income increased so did the consumption of SSB in children: more children consumed SSBs in all survey years, especially in the highest consumption groups (>2 times a week). The children who enjoyed eating fast food and salty snacks tended to show higher SSB consumption (>2 times a week) compared with the children who did not enjoy eating fast food and salty snacks (28.4% vs. 10.4% and 25.7% vs. 10.9% in 2004, 18.6% vs. 8.6% and 15.5% vs. 9.9% in 2006, 19.6% vs. 13.9% and 18.5% vs. 14.0% in 2009, and 30.3% vs. 19.3% and 27.1% vs. 21.3% in 2011). Meanwhile, children who spent more time on sedentary activities consumed more SSBs, particularly in the group ‘>2 times a week’. For sedentary time increasing from low to high, SSB consumption increased from 5.7% to 18.8% in 2004, from 10.1% to 16.8% in 2006, and from 14.8% to 20.5% in 2009, (Mantel-Haenszel p<0.01, for all three). Father and mother’s low education levels were associated with children’s higher SSB consumption (Mantel-Haenszel p<0.01). Moreover, the children whose parents consumed SSBs were inclined to have higher SSB consumption, P<0.01.

**Table 2:** Prevalence of SSB consumption in children in China from 2004 to 2011 (%).

	2004			2006			2009			2011		
	0/w	0–2/w	>2/w	0/w	0–2/w	>2/w	0/w	0–2/w	>2/w	0/w	0–2/w	>2/w
Total	27.4	58.4	14.2	24.7	61.9	13.4	12.9	69.4	17.7	9.7	68.5	21.8
Age												
6–11	26.5	58.8	14.7	23.9	62.7	13.4	12.5	68.8	18.7	9.5	70.6	19.9
12–17	28.1	58.1	13.8	25.5	61.1	13.4	13.3	70.1	16.6	9.9	65.8	24.3
Sex												
Boys	25.3	59.1	15.6	24.5	61.6	13.9	11.1	70.1	18.8	8.0	67.3	24.7 **
Girls	29.8	57.6	12.6	25.0	62.3	12.7	15.1	68.6	16.3	11.5	69.8	18.7
Residency												
Urban	16.0	60.4	23.6**	17.0	63.3	19.7**	10.1	63.7	26.2**	10.1	65.9	24.0
Rural	31.9	57.6	10.5	27.9	61.4	10.7	14.0	71.6	14.4	9.5	70.1	20.4
Education												
Primary school	30.8	57.5	11.7^##^	25.8	62.0	12.2	12.9	71.3	15.8	9.4	71.2	19.4^##^
Middle school	26.4	58.3	15.3	25.0	59.7	15.3	11.1	70.2	18.7	9.2	63.7	27.1
High school	15.4	67.7	16.9	15.9	65.1	19.0	13.5	75.7	10.8	6.7	61.3	32.0
Provincial categories												
Municipality	-	-	-	-	-	-	-	-	-	10.7	63.2	26.1**
Northeast	27.8	56.1	16.1**	23.0	59.2	17.8**	13.7	60.6	25.7**	7.7	66.2	26.1
East coast	23.4	58.2	18.4	13.3	61.7	25.0	15.9	59.5	24.6	9.4	59.4	31.2
Central	28.1	56.9	15.0	30.6	60.4	9.0	10.9	69.5	19.5	12.7	69.5	17.8
South	28.5	61.1	10.4	26.4	64.1	9.5	12.5	75.8	11.7	8.1	76.3	15.6
Household income												
Low	38.2	51.1	10.7^##^	35.3	56.3	8.4^##^	15.0	72.6	12.4^##^	11.3	71.5	17.2^##^
Medium	25.0	62.9	12.1	23.5	65.6	10.9	13.4	72.4	14.2	9.3	71.5	19.2
High	18.5	60.7	20.8	17.8	59.7	22.5	10.2	62.8	27.0	8.0	59.7	32.3
Like fast food												
Yes	11.8	59.8	28.4**	14.9	66.5	18.6**	6.8	73.6	19.6**	9.4	60.3	30.3**
No	31.7	57.9	10.4	33.7	57.7	8.6	19.2	66.9	13.9	10.3	70.4	19.3
Like salty snack food												
Yes	13.7	60.6	25.7**	19.1	65.4	15.5**	8.6	72.9	18.5**	7.7	65.2	27.1*
No	31.4	57.7	10.9	32.9	57.2	9.9	19.6	66.4	14.0	12.6	66.1	21.3
Like vegetables												
Yes	29.3	58.6	12.1	29.2	59.7	11.1**	15.5	69.6	14.9*	9.8	66.6	23.6
No	27.3	58.0	14.7	17.4	65.9	16.7	7.8	71.1	21.1	9.8	63.6	26.6
Like fruit												
Yes	26.9	59.0	14.1	26.2	61.3	12.5	12.8	70.4	16.8	9.0	67.7	23.3
No	29.4	57.4	13.2	24.3	61.4	14.3	16.7	67.8	15.5	14.2	54.7	31.1
Physical exercise outside of school												
Yes	20.8	60.1	19.0**	18.4	64.2	17.4**	9.9	70.6	19.6	6.9	67.1	26.0**
No	30.5	57.6	11.8	28.1	60.5	11.4	14.2	69.1	16.7	11.3	69.6	19.1
Physical exercise in school												
Yes	24.3	60.8	14.9	23.3	63.9	12.8**	12.0	70.2	17.8	7.9	69.9	22.2**
No	48.3	41.4	10.3	33.5	57.9	8.6	18.0	60.9	21.1	19.8	61.1	19.1
Sedentary activity												
low	39.1	55.2	5.7^##^	26.6	63.3	10.1^##^	25.3	59.9	14.8^##^	10.0	66.7	23.3^#^
Medium	24.6	60.0	15.4	26.2	62.1	11.7	11.4	71.7	16.9	11.5	69.5	19.0
High	19.0	62.2	18.8	21.1	62.1	16.8	10.4	69.1	20.5	7.0	68.9	24.1
Father’s education												
Primary and below	34.5	54.6	10.9^##^	27.4	62.3	10.3^#^	16.0	74.1	9.9^##^	11.0	74.9	14.1^##^
Middle school	25.1	58.7	16.2	24.7	60.5	14.8	11.8	68.8	19.4	8.1	68.3	23.6
Graduate and postgraduate	26.1	56.5	17.4	20.0	56.4	23.6	7.5	60.4	32.1	7.0	62.8	30.2
Father’s SSB consumption												
Yes	7.8	68.5	23.7**	11.5	68.8	19.7**	4.2	75.4	20.4**	4.6	70.4	25.0**
No	32.7	54.6	12.6	28.5	58.7	12.8	18.5	65.6	15.9	11.4	67.2	21.4
Mother’s education												
Primary and below	34.7	57.3	8.0^##^	30.6	62.3	7.1^##^	18.9	70.6	10.5^##^	11.4	75.3	13.3^##^
Middle school	22.2	58.9	18.9	21.9	61.3	16.8	9.4	69.4	21.2	8.6	66.7	24.7
Graduate and postgraduate	12.1	60.6	27.3	13.0	67.4	19.6	9.1	59.1	31.8	8.4	59.5	32.1
Mother’s SSB consumption												
Yes	10.1	68.4	21.5**	9.2	68.4	22.4**	3.1	74.3	22.6**	4.3	70.9	24.8**
No	35.6	53.1	11.3	31.6	58.9	9.5	24.0	63.6	12.4	15.8	63.8	20.4

* Chi-Square test: p<0.05;

** Chi-Square test: p<0.01;

^#^ Mantel-Haenszel test p<0.05;

^##^ Mantel-Haenszel test p<0.01.

### The effects of multiple factors on sugar-sweetened beverage consumption in children

The result of the multilevel mixed-effects logistic regression (Table 3) shows SSB consumption in children increased significantly from 2004 to 2011. The AOR increased from 0.96 (95% CI: 0.60–1.52) in 2006 to 2.32 (95% CI: 1.22–4.41) in 2011 compared with 2004. The model also shows during the same period, AOR for girls’ SSB consumption compared with boys was 0.83 (95% CI: 0.58–1.18), and for the consumption in rural areas compared with in urban was 0.79 (95% CI: 0.53–1.17). The SSB consumption of high school students was more than that of primary school students, AOR 2.13 (95% CI: 1.06–4.28). The children in high- and medium-income households had higher AOR of SSB consumption (1.62, 95% CI: 1.08–2.44 and 2.15, 95% CI: 1.26–3.68) than low-income households. The children who enjoyed eating fast food and salty snacks held the AOR: 1.52 (95 CI%: 0.96–2.40) and 1.49 (95% CI: 0.96–2.31). Children performing exercise out of school and in school consumed SSBs with AOR 1.22 (95% CI: 0.85–1.75) and 1.24 (95% CI: 0.67–2.27), respectively. Compared with low level of sedentarily activity, AORs for medium and high levels were 1.57 (95% CI: 0.99–2.49) and 1.89 (95% CI: 1.15–3.11). After adjusting for the factors involved in the logistic model, father and mother’s highest education levels were not statistically relevant to SSB consumption by their children, but parents’ SSB consumption was significantly linked to increased children’s consumption, AOR 3.83 (95% CI: 1.98–7.39) for father and AOR 5.54 (95% CI: 3.17–9.67) for mother.

**Table 3:** Multilevel mixed-effects logistic regression on SSB consumption and associated factors in children in China from 2004 to 2011.

	OR	95% CI	*P*
Wave (ref. = 2004)			
2006	0.96	(0.60–1.52)	0.8512
2009	1.29	(0.76–2.17)	0.3441
2011	2.32	(1.22–4.41)	0.0101
Age (ref. = 6–11)	0.31	(0.03–3.30)	0.3328
Sex (ref. = boy)	0.83	(0.58–1.18)	0.2967
Residency (ref. = urban)	0.79	(0.53–1.17)	0.2346
Education (ref. = primary school)			
Middle school	1.16	(0.81–1.65)	0.4262
High school	2.13	(1.06–4.28)	0.0345
Provincial categories (ref. = central)			
East coast	0.97	(0.55–1.70)	0.9017
Municipality	1.02	(0.35–2.97)	0.9742
Northeast	0.86	(0.53–1.40)	0.5416
South	0.94	(0.59–1.50)	0.7926
Household income (ref. = low)			
Medium	1.62	(1.08–2.44)	0.0198
High	2.15	(1.26–3.68)	0.0053
Like fast food (ref. = no)	1.52	(0.96–2.40)	0.0758
Like salty snack food (ref. = no)	1.49	(0.96–2.31)	0.0728
Like Vegetable (ref. = no)	0.68	(0.44–1.04)	0.0754
Like Fruit (ref. = no)	1.19	(0.76–1.88)	0.4490
Physical exercise off school (ref. = no)	1.22	(0.85–1.75)	0.2848
Physical exercise in school (ref. = no)	1.24	(0.67–2.27)	0.4944
Sedentary activity (ref. = low)			
Medium	1.57	(0.99–2.49)	0.0560
High	1.89	(1.15–3.11)	0.0119
Father’s education (ref. = primary and below)			
Middle school	0.90	(0.59–1.39)	0.6400
University and higher	0.60	(0.24–1.49)	0.2704
Father’s SSB consumption (ref. = no)	3.83	(1.98–7.39)	<0.0001
Mother’s education (ref. = primary and below)			
Middle school	1.06	(0.72–1.54)	0.7827
University and higher	0.89	(0.32–2.46)	0.8136
Mother’s SSB consumption (ref. = no)	5.54	(3.17–9.67)	<0.0001

Ref, reference category. AIC, 1141.91.

## Discussion

### Main findings

We found more than 80% of children in China reported consuming SSBs and the prevalence increased significantly from 2004 to 2011. The boys and the children who resided in urban areas, studied in high school, and lived in higher-income families tended to consume more SSBs than their respective counterparts. Children who enjoyed eating unhealthy food consumed more SSBs. Furthermore, physical activity and increased sedentary time, and parental SSB consumption were associated with raised prevalence of SSB consumption among children.

We found a high percentage of children in China reported SSB consumption, but the consumption frequency in this population was not high: approximately 83.2% of children consumed SSBs less than two times every week between 2004 and 2011. This finding was in accordance with previous research [23]. Compared with other countries, Chinese residents consumed a relatively low level of SSBs, but the increasing trend, particularly in children, was dramatic [30, 31]. This change mirrored the SSB sales in China issued by Euromonitor Passport International [14].

### Multi-domain of factors related to the upward trend of SSB consumption

We hypothesized that a multi-domain of factors related to the upward trend of SSB consumption in children in China. We found that girls consumed less SSBs than boys in the bivariate analysis. This difference can be attributed to the possibility that girls may care more about their body shape and were willing to keep fit by avoiding high energy-dense food and beverages [23, 32].

With the economy continuing to expand, China saw a significant lifestyle change among its residents. More and more people accepted highly processed food and beverages. Our research showed children who lived in urban areas consumed more SSBs in the bivariate analysis. One reason was that these areas had increasing emerging retailers, which boosted the dissemination of beverages to customers [33]. People with high accessibility to beverages would consume more than their counterparts [34, 35]. The other reason may be that these areas were socially and economically more developed than rural areas. The residents of these areas had high purchasing capability, and the beverage companies targeted their products to these people to earn more profit [36]. Furthermore, the beverage companies and retailers placed more importance on the high-income families, who had higher purchasing capability to consume SSBs [18, 37, 38].

Our research also showed children who preferred eating unhealthy food had a higher prevalence of SSB consumption. One of the reasons was those ultra-processed food providers expanded in the Chinese market, because of high profits attained from food industries. Those unhealthy foods also were targeted at the population with similar social economic background as SSBs. The food and beverage combination consumed by children led to increased health risks more than any one type of unhealthy food intake [26]. Research found that beverages were less likely than solid food to satisfy people’s appetite, even though these people had already consumed significant calories from SSBs [39, 40]. As a result, SSBs were less likely to reduce solid food consumption to maintain energy balance. People still can consume much processed food, such as fast food or salty food, after consuming a lot of SSBs. This increased the risk of obesity and related non-communicable diseases [30, 41].

Schools are the main living places where children’s health behavior and dietary pattern are cultivated. High school students usually had higher weekly allowances than middle and primary school students, and a major proportion of allowances were used to purchase beverages, so more high school students consumed SSBs [15, 42]. We also found that higher percentage of student consumed SSBs after physical activity both in school and out of school. Active children were inclined to consume beverages more frequently [19]. Also, peers influenced children’s dietary behaviors. The children who participate in physical activity together tended to share SSBs during exercise sessions [43, 44]. The availability of SSBs in corner stores or vending machines in schools and nearby neighborhoods increased SSB consumption [34].

In our research, sedentary activities were classified by time spent on homework plus screen activities. The children who spent more time performing sedentary activities consumed more SSBs. In China, approximately 80% of advertising on TV was for food and beverages [38], and so children with access to TV were exposed to these advertisements tempting them to purchase and consume more SSBs [16]. In addition, children were likely to consume SSBs when they were watching TV, as a way of lifting spirits, or a reward after completing their homework. Our research supported previous research that showed a positive association between sedentary activities and SSB consumption [28], although several other studies failed to find a significant relationship [38]. Our research demonstrated that parents had strong influence on SSB consumption by children. We found that children whose parents consumed SSBs had a higher incidence of consuming SSBs [45], and mothers showed the strongest impact (AOR 5.54) in this research [15, 44]. Besides parental dietary behavior, parental education levels were examined by previous researchers with regard to their impact on SSB consumption by children, but the results were conflicted. Some research conducted in western countries showed that lower parental education levels were related to increased SSB consumption by children, and higher parental education levels contributed to a reduction. One reason was that parents with higher education levels controlled their children’s access to beverages and limited the intake [33, 46]. Nevertheless, the percentages of reported SSB consumption in our research showed the inverse association that the parents with high education level may increase their children’s exposure to SSBs. These results were also agreed to by several other research studies [15, 29, 47]. The conflicted conclusion should be attributed to the different social environment. In western and high-income countries, SSBs were usually more affordable than in low- and middle-income countries [48], and parents with low education level tended to choose lower-priced SSBs to feed their children [17, 49–51]. While in China, SSBs actually are more expensive than bottled water. Parents with higher education status usually had good economic status [24] and were capable of buying SSBs for their children.

### Implications for decision making and practice

Implicated in our research, raising beverage prices may not be a sensitive and efficient measure to curb the increase of SSB consumption by children in China, because the majority of consumers actually had a high socio-economic background. This situation differs from that in western countries. Instead, prohibiting social marketing in urban and advanced areas, and communicating the associated health risks of SSBs to high income families, may potentially bring about improved health behavior and outcomes.

We used the data extracted from the CHNS, a national cohort study on nutrition and social economic development. The sample population was selected from the main Chinese territories where the majority of Chinese live. So, our research provided an overview of SSB consumption by children in China, which can inform health decision making. Our research comprehensively examined the trend of SSB consumption among children in China for eight years, when China experienced a swift change of lifestyle and dietary patterns, which boosted the knowledge body of this health issue. We also comprehensively explored several domains of factors linked to SSB consumption in children, including children’s demographics, economic background, residency, food preferences, physical activities, and parental influences. Resulting from more factors in the multilevel mixed-effects logistic regression, we accurately identified the odds ratio of these factors for the SSB consumption by children in China. Because larger AORs demonstrate a stronger relationship between SSB consumption and factors, we can prioritize factors that may strongly influence SSB consumption and target them when making health strategies.

### Limitations

This research used the data from self-reported food frequency questionnaires. The amounts of SSB consumption by children cannot be accurately measured and, as a result, we did not examine the dose-response effects between SSB consumption and those factors. Furthermore, as a descriptive quantitative research, our research was limited to deeply narrate the mechanism and pathway of the factors affecting SSB consumption in children.

Further studies are needed to employ precise measures on the amount of SSB consumption and the factors among children. In addition, mixed methodology combining quantitative survey and qualitative investigation may enhance the validity of this health research. Moreover, by conducting qualitative research, in-depth insights into occurrence, development, and consequence of SSB consumption by children can be gained.

## Conclusions

In conclusion, our research illustrates that SSB consumption in children in China increased significantly from 2004 to 2011. Promising measures to reduce SSB consumption are urgently needed. By combining the efforts from parents, neighborhoods, and the community, a planned reduction of SSB consumption aimed at the children with high economic background, increased sedentary time, and high parental SSB consumption will help to curb the increase of obesity and NCDs among children in China.
